# Editorial: Antimalarial chemotherapy in the XXIst century, volume II

**DOI:** 10.3389/fphar.2023.1229764

**Published:** 2023-06-30

**Authors:** Paula Gomes, Rafael V. C. Guido

**Affiliations:** ^1^ LAQV-REQUIMTE, Department of Chemistry and Biochemistry, Faculty of Sciences (FCUP), University of Porto, Porto, Portugal; ^2^ São Carlos Institute of Physics, University of São Paulo, São Carlos, São Paulo, Brazil

**Keywords:** malaria, drug discovery, inhibitor, plasmodium, antimalarial agents

The Global Technical Strategy for malaria (GTS) (World Health Organization, 2015) was introduced by the World Health Organization (WHO) Assembly in May 2015. The document presented an inclusive framework aimed at assisting countries in accelerating their advancements towards malaria elimination. In this sense, the Assembly established several targets for the GTS by 2030 including: 1) reduce malaria incidence and mortality rates globally (at least 90%); 2) eliminate malaria from countries in which malaria is transmitted (at least 35 countries); and 3) prevent re-establishment of malaria in all countries that are malaria-free. To do so, heavy investments and efforts should have been done to deliver new tools within the lifetime of this strategy. These tools included new and more effective medicines, new combinations of medicines, improved diagnostics, new vaccines, new insecticides and other innovative vector control methods (World Health Organization, 2015). The 2021 update of GTS provided estimates of the funding required to achieve key targets in 2030 (World Health Organization, 2021). The revised figure highlighted that in order to achieve an 80% or higher coverage of current interventions, a substantial investment in malaria research and development (R&D) should increase from US$ 3.1 billion (2021) to US$ 10.3 billion (2030). Consequently, these investments would deliver new malaria tools to tackle emerging challenges like urban malaria and the proliferation of antimalarial drug resistance (World Health Organization, 2022).

The development of novel products for malaria treatment and prevention relies on several strategies including: 1) innovations with existing antimalarial treatments (e.g., triple artemisinin-based combination (ACT) therapy, ACT plus single low-dose primaquine for transmission blocking, and artemether-lumefantrine for neonates); 2) next-generation antimalarial treatments (ganaplacide-lumefantrine, M5717-pyronaridine, ZY19489-ferroquine, and cipargamin—formerly known as KAE609); 3) candidate molecules in early development (MMV533; INE963, GSK701, MMV183, GSK484, IWY357, and MMV609), and 4) next-generation chemoprevention treatments [MMV371—a prodrug of atovaquone with high liver-stage potency and MMV167 (ELQ-331) a prodrug of ELQ300] (Cabrera, 2019; Schalkwijk et al., 2019; Smilkstein et al., 2019; Medicines for Malaria Venture, 2021; Murithi et al., 2021; Taft et al., 2022; Bopp et al., 2023). The Medicines for Malaria Venture (MMV) (Medicines for Malaria Venture, 2023), a non-profit organization, has provided crucial support for the discovery and development of these drug candidates. MMV collaborates with a diverse network of international organizations, academic institutions, public-sector entities, non-governmental organizations (NGOs), and private-sector entities. Together, they are dedicated to conducting R&D in malaria medicine, with a primary focus on discovering and enhancing accessibility to the next-generation of life-saving drugs. This collaborative effort is particularly aimed at benefiting adults, including pregnant women, and children who face an increased risk of malaria-related complications.

Aligned with the GTS and the worldwide efforts to discover and deliver new antimalarial treatments, the Research Topic “*Antimalarial Chemotherapy in the 21st Century - Volume II*” presents a compilation of three original articles and one review focusing on the challenges related to the pharmacodynamics, pharmacokinetics, and formulations of novel antimalarial candidates. One of the articles by Cólon-Lorenzo et al. explored glutathione S-transferase (GST), a crucial enzyme in the glutathione pathway, as a potential molecular target for malaria treatment. Their study sheds light on the role of GST in parasites and assessed its viability as a target for drug development. Using reverse genetics, they provided experimental evidence that GST is essential for the survival of *Plasmodium berghei* intra-erythrocytic forms. Additionally, they employ a structural model of the enzyme to screen a library of 900,000 compounds. CB-27, one of the compounds identified, exhibited significant inhibitory activity (in the submicromolar range) against multidrug-resistant strains. Through shape similarity screening using CB-27 as a reference, new chemical scaffolds with potent antiplasmodial activity and desirable drug-like properties were discovered. Kumar et al. investigated the antiplasmodial activity of thymol derivatives against chloroquine-sensitive and chloroquine-resistant strains of *P. falciparum*. Their mode-of-action studies revealed that 4-chlorothymol increased the levels of reactive oxygen and nitrogen species while altering the redox balance by affecting the activity of GST and glutathione reductase (GR) enzymes. *In vivo* experiments with a murine model infected with *Plasmodium yoelii nigeriensis* showed promising activity of 4-chlorothymol, resulting in reduced parasitemia and improved survival. When combined with chloroquine, 4-chlorothymol demonstrated enhanced chemosuppression and extended mean survival time compared to individual doses of chloroquine and 4-chlorothymol. These findings indicated that 4-chlorothymol is a potential lead compound for antimalarial discovery. Watson et al. investigated the pharmacokinetic and physicochemical properties of decoquinate (DQ) derivatives, a chemical series exhibiting potent activity against *P. falciparum* and potential suitability as a non-artemisinin partner for combination therapy. However, DQ itself exhibited unfavorable drug-like properties such as high lipophilicity and poor water solubility. To address these issues, modifications were made to DQ, resulting in new derivatives (RMB005, RMB059, and RMB060). The *in vitro* and *in vivo* absorption, distribution, metabolism, and excretion (ADME) properties of these derivatives were assessed. The amide derivative RMB005 was designed to have improved physicochemical properties compared to DQ. However, the ADME studies revealed that RMB005 exhibited low solubility and permeability, leading to limited bioavailability. Similarly, the carbamate derivatives RMB059 and RMB060 were expected to demonstrate improved properties compared to RMB005, but they proved to be highly unstable under physiological conditions, rapidly converting to DQ *in vivo*. This work emphasizes the significance of conducting comprehensive ADME investigations to accurately assess the potential of drug candidates. Finally, Chaves et al. presented a review focusing on the application of nanotechnology in preclinical formulations of antimalarial agents. They discuss the impact of these approaches in advancing antimalarial candidates to clinical studies.

This Research Topic highlights the use of experimental and computational methods in drug discovery and development to design novel antimalarial agents and formulations. With three original articles and one review, it offers additional insights into the challenges and limitations of antimalarial chemotherapy in the XXIst century.
